# Epstein–Barr virus dynamics and its prognostic impact on nasopharyngeal cancers in a non-endemic region

**DOI:** 10.3332/ecancer.2022.1479

**Published:** 2022-12-02

**Authors:** Rajitha Ganapathi, Rejnish Ravi Kumar, Kainickal Cessal Thomas, Malu Rafi, Kannan Sankara Reddiar, Preethi Sara George, Kunnambath Ramadas

**Affiliations:** 1 Department of Radiation Oncology, Regional Cancer Centre, Trivandrum, Kerala 695011, India (Current Address: Department of Health Services, Government of Kerala, Kerala 695035, India); 2 Division of Cancer Research, Regional Cancer Centre, Trivandrum, Kerala 695011, India; 3 Department of Cancer Epidemiology and Biostatistics, Regional Cancer Centre, Trivandrum, Kerala 695011, India; 4 Department of Radiation Oncology, Regional Cancer Centre, Trivandrum, Kerala 695011, India (Current address: Director - Radiation and Allied Services, KARKINOS, Ernakulum, Kerala 682017, India)

**Keywords:** nasopharyngeal cancer, non-endemic region, EBV DNA dynamics, EBV serology, factors affecting treatment outcomes

## Abstract

**Background:**

Epstein–Barr virus (EBV) DNA quantification in nasopharyngeal cancer (NPC) is an indicator of the tumour burden, stage and survival. Although EBV dynamics in endemic regions has been extensively studied and reported, the data from non-endemic regions is sparse. This study attempts to investigate the EBV dynamics in NPC patients from a non-endemic region and also to identify the factors impacting the outcomes.

**Materials and methods:**

This was a prospective observational study conducted at a tertiary care centre in South India and enrolled patients with non-metastatic, biopsy proven NPC, who were suitable for radical chemo-radiotherapy with or without induction chemotherapy. Two blood samples, one prior to initiation of any anticancer treatment, and second at 6 weeks post treatment, were collected to quantify EBV DNA using real-time quantitative polymerase chain reaction. Antibodies against EBV viral capsid antigen (EBV VCA IgM), EBV Early Antigen (EBV EA IgG) and EBV Nuclear Antigen (EBV EBNA IgG) were also measured in the sample. The impact of EBV dynamics on the outcomes was then analysed.

**Results:**

The study included a total of 35 patients. Thirty-three had identifiable EBV DNA (94.3%) and a histological diagnosis of non-keratinising undifferentiated type of squamous cell carcinoma. There was no correlation between the EBV DNA and anti-EBV antibodies. There was a significant association between composite stage and pre-treatment DNA titre (*p* = 0.030). The mean EBV DNA titre was lower for patients with no clinically demonstrable disease at last follow-up and the reduction in EBV DNA titres was significant (*p* = 0.020) for those patients who remained disease free.

**Conclusion:**

Plasma EBV DNA is an accurate and reliable biomarker for NPC for WHO type 2 and 3 tumours even in non-endemic regions.

## Background

Nasopharyngeal cancers (NPCs) differ from other head and neck squamous cell carcinomas in unbalanced geographical distribution [1], multifactorial origin, association with Epstein–Barr virus (EBV) as well as clinical behaviour. The association of EBV with NPC is well established in the endemic regions where the EBV dynamics has been extensively studied and reported. EBV is known for its dual tropism for human B-cells and nasopharyngeal epithelial cells, where they can establish latent infection and subsequently integrate with the host genome. Expression of various EBV-encoded latent genes can lead to growth and progression of EBV infected pre-malignant cells to undifferentiated cancers. This is potentiated by presence of cofactors such as an inflammatory micro-environment, alterations in cell-cycle regulatory mechanisms like inactivation of p16 and over expression of cyclin-D1 [2, 3].

Various viral markers and antibodies against viral components have been used for detection of EBV infection, as a marker for assessing tumour burden and as a tool for monitoring treatment response. EBV DNA quantification, which is a direct reflection of the viral load, is an indicator of the tumour load, stage and survival for NPC patients [4–7]. This can be done using quantitative polymerase chain reaction (PCR) on tumour tissue or body fluids like the plasma and saliva [8, 9]. Evidence, however, suggests that salivary assays may not be very accurate [10] when compared to plasma assays. The role of EBV DNA in prognostication and subsequent treatment modification is an ongoing research avenue with many promising leads published so far. Multiple EBV specific antibodies like immunoglobulin A (IgA), antibody against viral capsid antigen (VCA-IgA) have been demonstrated in patients with EBV associated NPC [11–14]. Investigations to prove the potential use of these antibody assays as a cheap and easier surrogate for the cumbersome DNA study have been mostly futile [15].

This study attempts to investigate the EBV dynamics in NPC patients, by the absolute quantification of EBNA-1 gene in EBV genome, from a non-endemic region and also identify the factors impacting the outcomes. A previous study undertaken at the institute has reported an EBV DNA prevalence of 63% [16] among NPC patients.

## Methods

This was a prospective observational study conducted at a tertiary care centre in South India. The study was conducted after obtaining the necessary institutional regulatory approvals (Scientific committee functioning in the institution sanction – IRB no 12/2015/09 and Human Ethics Committee – HEC no: 02/2016).

The study enrolled patients with non-metastatic, biopsy proven NPC, who were treated at the institute between February 2016 and June 2017 and willing to give an informed consent. Only those evaluated and found suitable for radical chemo-radiotherapy were included. All patients uniformly underwent endoscopic evaluation, radiological evaluation for staging and metastatic workup [computed tomography (CT), magnetic resonance imaging (MRI) or positron emission tomography (PET)-CT] pre-radiotherapy assessments and the baseline blood workup. Staging was done as per American Joint Committee on Cancer (AJCC) 7th edition criteria. Patients with bulky nodal disease or locally advanced disease in close proximity to critical structures were given neo-adjuvant chemotherapy (PF regimen) for disease down-staging prior to radiotherapy (RT). RT was delivered using RapidArc intensity modulated radiotherapy technique (IMRT) technique using 6 MV photon and simultaneous integrated boost schedule [66, 60 and 54 Gy in 30 fractions to planning target volume (PTV) 1 2 and 3, respectively] along with concurrent 3 weekly cisplatin or carboplatin.

The first post treatment clinical evaluation was done at 2 months. Endoscopy and imaging for response assessment, as per the Response Evaluation Criteria in Solid Tumors v1.1, were done at 3 months. Overall survival (OS) and disease free survival (DFS) were calculated from the date of diagnosis. All patients were followed up at 3 monthly intervals for 2 years and 6 monthly intervals thereafter until December 2021.

### EBV estimation

Blood samples were collected just prior to initiation of any anticancer treatment, either neo-adjuvant chemotherapy or chemo-radiotherapy, and at 6 weeks post RT. The first sample was separated into serum and plasma for antibody assay and DNA quantification studies and only DNA studies were done on the second sample. The plasma sample was subjected to a two-step enzyme immune assay for detecting antibodies against EBV viral capsid antigen (EBV VCA IgM), EBV Early Antigen (EBV EA IgG) and EBV Nuclear Antigen (EBV EBNA IgG).

Plasma DNA was extracted using a QIAamp DNA Blood Mini-Kit (Qiagen, Hilden, Germany) and EBV copy number was quantitated using the RealStar EBV PCR Kit 1.0 (Altona Diagnostics GmbH, Hamburg, Germany) based on real-time PCR (RT-PCR) approach. The RealStar EBV PCR Kit 1.0 is a CE-marked diagnostic kit according to the European in vitro diagnostic directive 98/79/EC. The kit contains primer-probes specific to the EBNA-1 gene in the EBV genome, master mix and four quantification standards to create the standard curve to quantitate the EBV load in the plasma samples. These Quantification Standards were calibrated against the 1st World Health Organization International Standard for Epstein–Barr Virus for Nucleic Acid Amplification Techniques [National Institute for Biological Standards and Control (NIBSC) code: 09/260]. Probes specific for EBV DNA are labelled with the fluorophore FAM™. The assay was done as per the protocol provided by the kit manufacturer. The EBV load is expressed in IU/??L units. The lower and upper limits of concentration of EBV specific DNA in the samples analysed were set as 0 and 5,000 IU/mL, respectively. Since we measured the EBV load by real-time quantitative polymerase chain reaction (qRT-PCR) using absolute quantification approach, we expressed the EBV load in International Units per microlitre (IU/??L). We have dichotomised the patients based on the EBV load with a cut of point 500 IU as </=500 and >500.

### Statistical analysis

The prevalence of EBV in the study population was reported using descriptive statistics. Statistical analysis was done using SPSS software and all reported *p* values are two-sided. Statistical significance was set at *p* value < 0.05.

Correlation between Anti-EBV antibody titres and pre-treatment DNA was assessed using Pearson’s correlation. Paired t test was used to assess the difference in pre and post treatment EBV DNA titres using a cutoff value of 500 IU/mL. Patients were divided into two groups based on their pre-treatment values (</=500 IU/mL and >500 IU/mL) and were divided into three groups based on their post treatment values (undetectable values after 40 cycles of qRT-PCR; up to 500 IU/mL and up to 5,000 IU/mL). Chi2/Fisher’s exact test was used to analyse the association between EBV DNA load (pre-treatment and post treatment) and various clinical parameters like composite stage. The association between dynamics of EBV DNA with disease status at 3 months, status at 12 months and end of follow-up was assessed using Mann–Whitney *U* test. Kaplan–Meier method was used to assess the cumulative OS probability and DFS probability. The survival outcomes with respect to DNA titres were analysed using log rank tests after stratifying patients into two groups – with EBV titre cutoff at 1,000 IU/mL.

## Results

The study included a total of 35 patients with non-metastatic, biopsy proven NPC who were followed up till December 2021, with a median follow-up of 42.6 months (range: 3.6–48.7). Sixty percentage of the patients presented with locally advanced disease and more than 90% (94.3%) had histological diagnosis of non-keratinising undifferentiated type of squamous cell carcinoma. Induction chemotherapy was used in 27 (77.1%) and 37 patients (97.1%) received concurrent chemotherapy. 88.6% of patients completed RT without any interruptions and 71.4% received two cycles of concurrent chemotherapy. Two patients were given concurrent carboplatin due to cardiac disease and diabetic nephropathy. Two patients discontinued treatment after the first cycle of concurrent chemotherapy due to unknown reasons. Concurrent chemotherapy had to be deferred by 1 week in one patient who developed grade 3 mucositis, dermatitis and grade 4 neutropenia (Table1).

### Prevalence of EBV

Pre-treatment EBV DNA titres were available for all 35 patients. The post treatment reports were however available only in 31 patients, after excluding two patients who discontinued treatment and one patient who did not turn up for the scheduled follow-up. Among the 35 samples tested for EBV DNA at diagnosis, 33 had identifiable EBV DNA (94.3%). One patient with undetectable EBV DNA had undifferentiated carcinoma and the histology for the second patient was well differentiated keratinising squamous cell carcinoma.

### EBV DNA and anti-EBV antibodies

Antibody titres (EBV VCA IgM, EBV EA IgG and EBV NA IgG) were estimated in the sera of 34 patients. It was not done in one patient due to sample inadequacy. There was no significant correlation between the EBV DNA and anti-EBV antibodies (Table 2).

Association between pre-treatment DNA titre and variables like sex, tumour (T) status, nodal (N) status, composite stage, residual disease, recurrence, disease status at 6 and 12 months post treatment was assessed. There was significant association between composite stage and pre-treatment DNA titre (Table 3). Those patients with locally advanced disease had titres of >500 IU/mL and all five patients who were stage II had titres < 500 IU/mL.

There was no statistically significant correlation between the post treatment DNA titre and the treatment response at 3 months (*p* = 0.085), even though the mean EBV DNA titre was lower for patients with no clinically demonstrable disease at 6, 12 months and at last follow-up (Table 4).

Patients were stratified into three based on the end of treatment DNA titres and the association with disease status at various follow-up time points was evaluated. None of the patients with undetectable DNA titre after treatment had disease recurrence (Table 5).

The change in EBV DNA titres after treatment was analysed separately for patients who were disease free at last follow-up and those who had recurrence/died due to disease. The reduction in EBV DNA titres was significant (*p* = 0.020) for those patients who remained disease free (Table 6).

### Survival outcomes

The cumulative 2 year OS was estimated to be 88.2% [standard error (SE): 0.055] and DFS was 82.4% (SE: 0.065). The survival was stratified according to the pre and post treatment EBV DNA values and patients with pre and post treatment titre more than 1,000 IU/mL had poor OS and DFS although only the impact of the post treatment titres was statistically significant (Table 7).

**Figure 1.**
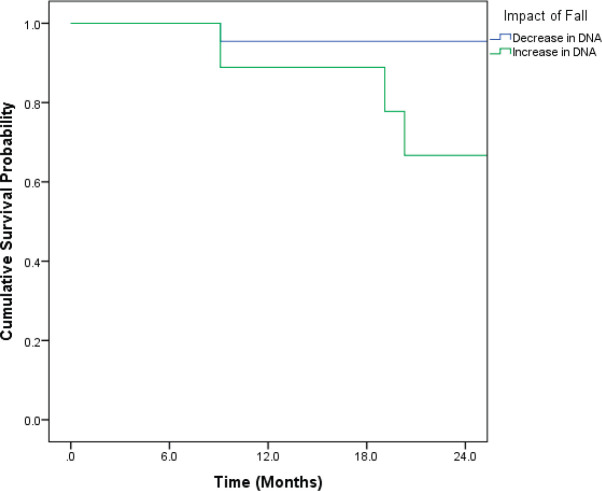
Kaplan–Meir curve showing the impact of change in DNA titre on OS (*p* = 0.091*).

**Figure 2.**
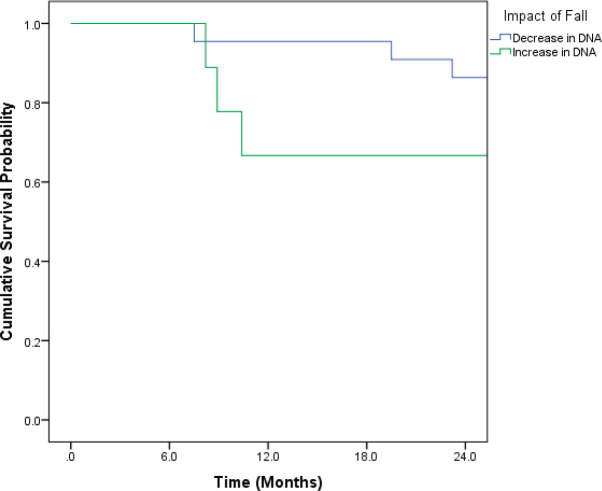
Kaplan–Meir curve showing impact of change in DNA titre on DFS (*p* = 0.267).

Patients who recorded an increase in the DNA titres over the course of follow-up had inferior OS and DFS. Even though the differences were pronounced, they did not attain statistical significance (Figures 1 and 2).

## Discussion

The daunting issue with NPC is that the majority of patients present in locally advanced stages and there is a paucity of recommendations for tailoring treatment according to specific biomarkers. Identification of sensitive and accurate biomarkers could pave way for prognostication, optimising treatment outcomes and in follow-up, for early detection and radical treatment of relapses.

Even though earlier studies tested the role of EBV related serology, this has been largely abandoned in favour of plasma EBV DNA studies following the discovery that the viral genome is released into the host circulation in EBV associated NPC [8, 17–20]. Thereafter, many studies were undertaken to evaluate the role of plasma EBV DNA in screening, prognostication and individualising treatment. The screening studies were almost entirely limited to the endemic regions [21–25].

The association between EBV related NPC and histological diagnosis of WHO type 2/3 cancers is well established and subsequently these subtypes predominate the case pool found in the endemic regions [26]. The limited information on the EBV dynamics from non-endemic regions suggests that EBV prevalence in NPC is high in these areas and points to the predictive role of the viral load at diagnosis to the stage and treatment outcomes [10, 27]. An EBV prevalence of more than 60% in NPC patients has been reported from the Institute previously [16] and correspondingly more than 90% of the patients in this study had a histological diagnosis of non-keratinising undifferentiated type of squamous cell carcinoma.

Multiple EBV associated antibodies have been demonstrated in the sera of NPC patients and these were tested for use in screening and early detection [13, 28]. Considering the relative ease of performing an antibody assay, we tested for any correlation between the EBV associated antibodies and DNA titres and in concordance with most other studies we could not demonstrate any correlation.

Although several studies and meta-analysis have been equivocal on the prognostic role of EBV DNA in predicting the stage and outcome of these patients [29–31], there is little consensus on the cut-off values for EBV DNA as a prognostic factor. Lin *et al* [32] and Wang *et al* [33] observed that <1,500 copies/mL EBV DNA before treatment were predictive of better OS. A similar cut off value of 2,000 copies /mL has been reported by Guo *et al* [34]. A much higher limit of 8,000 copies/mL was used by Chai *et al* [35], whereas an intermediate cutoff of 4,000 copies/mL was proven to be predictive of outcome by Hou *et al* [18]. Lai *et al* [36] performed a meta-analysis and reported that patients with pre-treatment EBV DNA load of more than 4,000 copies/mL had better OS with the addition of induction chemotherapy. In a study reported from Morocco, patients with a high pretreatment EBV DNA levels (≥ 2,000 IU/mL or ≥ 4,000 IU/mL) had significantly lower OS at 3 years (*p* < 0.05) [28]. They also found that patients with EBV DNA levels less than 1,500 IU/mL had inferior outcomes although the difference was not statistically significant (OS at 3 years: 84% versus 61%; *p* = 0.08)

We have reported the DNA assay in IU/mL and the conversion factor of 0.62 may be used for converting to copy numbers/mL [37]. A smaller cut off value of 500 IU/mL (746.26 copies/mL) was found to be predictive of the stage of disease in this study and all the patients with stage II disease in the cohort had a DNA titre < 500 IU/mL. A higher pre-treatment cutoff value of 1,000 IU/mL was found to be predictors of survival outcomes.

The clearance of EBV DNA is postulated to be a reliable predictor of outcomes after RT [38] and early responders after induction chemotherapy who also showed a rapid fall in the EBV DNA concentrations fared better than the late responders [39]. Even though more than 70% of patients were given induction chemotherapy in this study, interim DNA studies were not included in the protocol and hence the rate of fall of DNA titres could not be estimated. However, the patients who remained disease free at last follow-up was found to have a significantly higher mean fall in EBV DNA titre compared to those who had active disease. On the other hand, the patients who had an increase in the DNA titre had inferior DFS and OS.

The predictive role of detectable post treatment EBV DNA is also well proven [40–42] and this has paved way for biomarker guided, risk adapted treatment protocols. Even though the study by Chan *et al* [43] failed to show benefit for risk adapted treatment for patients with elevated plasma EBV DNA levels at 6–8 weeks post RT, there are several ongoing trials like the NCT04072107 study which may usher in new paradigms in risk adapted management. The ideal time for post treatment evaluation remains unknown, with several investigators choosing 6–8 weeks post treatment, for EBV testing. Lee *et al* [41] found that patients with elevated EBV DNA levels at 8 weeks after treatment had complete clearance on further follow-up. We have done a single post treatment evaluation of the EBV titres at 6 weeks following completion of RT and found that patients with post treatment titres < 1,000 IU/mL had a significantly better OS and DFS and all the patients who remained disease free till the last follow-up had undetectable post treatment titres. This further highlights the fact that adjuvant treatment may be safely omitted in good responders and opens avenues to test the potential for using post treatment titres for tailoring treatment, especially in considering adjuvant systemic treatment.

These finding support the prognostic importance of EBV DNA in staging and outcome prediction even in the non-endemic population.

### Limitations of the study

The main limitation of the study is the small sample size. The findings of this study may be considered as a stepping stone for further research in this direction in non-endemic regions.

## Conclusion

This study has demonstrated a positive association of EBV DNA in diagnosis and prognostication of NPC, especially WHO type2 and 3 tumours, in a non-endemic region. There was no correlation found between EBV DNA and EBV associated antibodies. Pretreatment DNA titres were predictive of the tumour burden and post-treatment titres were reflective of the treatment response and outcomes.

## Conflicts of interest

None.

## Funding

There are no financial conflicts of interest to disclose in the conduct of the study by any of the authors.

## Figures and Tables

**Table 1.** Demographics and treatment details.

Sex	
Male	29
Female	6
Stage	
II	5
III	9
IV-A	12
IV-b	9
Histology	
Non-keratinising undifferentiated	33
Keratinising SCC	1
Non-keratinising SCC	1
Chemotherapy	
Induction	27
Concurrent	34

**Table 2.** Co-relation between pre-treatment DNA and EBV antibodies.

Pre-treatment DNA	EBV NA_IgG	EA-IgG	VCA-IgM
Pearson correlation	0.140	0.010	−0.067
Sig. (2-tailed)	0.429	0.954	0.709

**Table 3.** Pre-treatment EBV DNA and disease stage.

Stage	Pre-treatment DNA levels		*N* = 35	*p* value
	<500 IU/mL	>500 IU/mL		
II	5		5	0.030*
III	3	6	9	
IVA	3	9	12	
IVB	3	6	9	
*N* = 35	14	21	35	

**Table 4.** Mean DNA titre and disease status at follow-up.

Time of follow-up/disease status	Mean DNA titre in IU/mL (standard deviation)		*p* value
	NED	Disease+	
6 months	438.5 (1193.8)	2,886.0 (2426.4)	0.002**
12 months	483.6 (1236.8)	3090.3 (2399.9)	0.005**
Last follow-up	432.4 (1194.8)	2598.2 (2270.4)	0.002**

NED: no evidence of disease

**Table 5.** Disease status according to post-treatment DNA stratification.

Follow-up	Undetectable		0 to </=500 IU/mL		>500 to 5,000 IU/mL		*p* value
	NED (*n*)	Disease + (*n*)	NED (*n*)	Disease + (*n*)	NED (*n*)	Disease + (*n*)	
6 months	16	0	6	2	3	4	0.003**
12 months	13	0	6	1	4	2	0.103
LFU	16	0	5	2	3	4	0.011*

NED: no evidence of disease; LFU: last follow-up

**Table 6.** Change in titre and status at last follow-up.

Status at last follow-up	Pre-treatment DNA IU/mL (mean)	Post-treatment DNA IU/mL (mean)	*p* value
NED - *n* (SD)	1,717.04 (2004.9)	453.9 (1,214.0)	0.020*
Disease +- *n* (SD)	3,210.5 (1,394.3)	2,395.7 (2,401.8)	0.486

NED: no evidence of disease

**Table 7.** Pre and post-treatment DNA and survival.

Pre-treatment EBV DNA titre IU/mL				
**Value**	**OS % (SE)**	***p* value**	**DFS% (SE)**	***p* value**
<=1,000	94.1 (0.057)	0.341	88.2 (0.078)	0.188
>1,000	82.4 (0.092)		76.5 (0.103)	
Post-treatment EBV DNA titre IU/mL				
<=1,000	100.0	<0.001*	92.0	0.002*
>1,000	33.3 (0.192)		33.3 (0.192)	
